# Associations between physical fitness and executive functions in 10–13 year male children in Saudi Arabia

**DOI:** 10.3389/fpsyg.2025.1493206

**Published:** 2025-03-26

**Authors:** Rana Jamaan Alghamdi, Salem Alotaibi, Osama Aljuhani, Shaea Alkahtani

**Affiliations:** ^1^Department of Psychology, King Saud University, Riyadh, Saudi Arabia; ^2^Department of Exercise Physiology, College of Sport Sciences and Physical Activity, King Saud University, Riyadh, Saudi Arabia; ^3^Department of Physical Education, College of Sport Sciences and Physical Activity, King Saud University, Riyadh, Saudi Arabia

**Keywords:** cognitive functions, physical fitness, preadolescents, working memory executive, schoolchildren

## Abstract

**Objectives:**

The aim of the present study was investigating the associations between physical fitness and executive function tasks in children aged 10–13 years, with a particular focus on the contribution of physical fitness to working memory.

**Findings:**

The findings reported significant correlations between the N-Back task (a measure of working memory) and all three physical fitness tasks: grip strength, long jump, and shuttle run. Additionally, the Flanker task (assessing selective attention) was correlated only with the shuttle run task. However, no significant correlation was found between the Stroop task (measuring inhibition control) and the physical fitness tests. Regression analyses further demonstrated that physical fitness tasks significantly contributed to working memory performance, independent of age and BMI.

**Contributions:**

These findings emphasize the importance of incorporating diverse physical activities in children’s routines to support both physical and cognitive development, particularly in enhancing working memory, which is critical for academic achievement. From an educational and policy standpoint, integrating targeted fitness programs into school curricula may foster both physical health and cognitive development. Consequently, this study underscores the need for holistic approaches that combine physical and cognitive interventions, ultimately enhancing overall development and academic performance in schoolchildren. Hence, this study underscores the need for holistic educational interventions that integrate physical fitness with cognitive development.

## Introduction

Motor skills are defined as the abilities required to control and coordinate the body’s movements, essential for daily activities and various forms of physical exercise (Bolger et al., 2021; Dapp et al., 2021). These skills are typically categorized into gross motor skills, which involve large muscle groups and whole-body movements, and fine motor skills, which involve smaller muscle groups and precise movements, such as those required for writing or manipulating small objects (Cerit et al., 2020; Gallahue, 2010). In the early childhood such as at pre-school and early primary school, the development of motor skills is crucial during childhood as it not only influences physical health but also plays a crucial role in social interaction and cognitive development including executive functions (Donnelly et al., 2016; Han et al., 2022; Van der Fels et al., 2015). In the late childhood and early adolescents, there is more intention to improve physical fitness. Physical fitness means the components of physical fitness including respiratory and muscular fitness, which are related to physiological, psychological and cognitive development including executive functions (Caspersen et al., 1985; Nieto-López et al., 2020).

Executive functions as an umbrella term (Diamond, 2013a; Mulder et al., 2009) is now associated with goal-directed behavior, encompassing multiple cognitive components such as inhibition control, selective attention, and working memory. Inhibition control is well known as the ability to control impulsive responses (Allom et al., 2016). Selective attention is typically defined as the ability to focus on selected target stimuli while ignoring other irrelevant stimuli (Lamers et al., 2010). Working memory, which is often defined as the cognitive function responsible for retaining and manipulating information in memory for a short period of time, and it is a core component of executive functions (Gray et al., 2017; Lee et al., 2013; Miyake et al., 2000). The development of executive functions during childhood is critical for academic achievement, social functioning, and overall mental health (Diamond, 2013a, 2013b; Nayfeld et al., 2013). These cognitive processes are primarily governed by the prefrontal cortex, which continues to develop throughout childhood and adolescence (Coull et al., 1996; Uttal et al., 2013).

The specific nature of the relationship between physical fitness and various components of executive functions remains underexplored, particularly in children aged 10–13 years. This age range is critical as it encompasses the transition from middle childhood to early adolescence, a period marked by significant cognitive, physical, and emotional development (Borghese and Janssen, 2019; Calvert et al., 2001; Pavlović et al., 2020; Vieira et al., 2003). Understanding the interactions between physical fitness and executive functions during this period can provide insights into how these domains support each other and inform interventions that promote holistic development. A growing body of literatures have indicated that physical activity enhances and facilitates neurocognitive performance especially children’s executive function (Amatriain-Fernández et al., 2021; Niederer et al., 2011; Pontifex et al., 2014; Tomporowski et al., 2008). One of the primary mechanisms through which exercise influences cognitive development is its impact on brain health (Amatriain-Fernández et al., 2021; Erickson et al., 2014; Meijer et al., 2021). Aerobic exercise, in particular, has been linked to increased gray matter volume in regions responsible for executive functions and memory (Chaddock-Heyman et al., 2014a,b; Erickson et al., 2014; Erickson et al., 2011; Weinstein et al., 2012). This structural plasticity is thought to underlie improvements in cognitive abilities. A recent systematic review (Van Waelvelde et al., 2020) concerning the associations between physical activity PA and cognitive performance confirmed the positive correlations between PA and certain measures of EFs such as Flanker and Stroop (Dallaway et al., 2023).

The conflicting research results regarding the association between physical fitness and executive function is an important rational of our paper. For example, among children aged 8–13 years, only handgrip strength was significantly associated with cognitive function, whereas other physical fitness components were not which could be due to the poor level of physical fitness among the majority of children participants (Amenya et al., 2021). Among adolescents, speed-agility was significantly associated with some cognitive functions, but cardiorespiratory and muscular fitness were not (Haverkamp et al., 2021). On the other hand, another study found an association between physical fitness components and four cognitive functions although these associations were mostly small (Drozdowska et al., 2021). Although there is evidence to suggest that there are positive associations among physical activity, fitness, cognition, and academic achievement, there findings are inconsistent. Even if there is conflicting outcomes regarding physical activity, it is important for growth and development and general health (Donnelly et al., 2016). Hence, this study aimed to investigate the associations between physical fitness tests and three measures of executive functions namely Stroop, Flanker and N.Back that assess inhibition control, selective attention and working memory, respectively. The purpose of this study also was to examine the contribution of physical fitness tests on executive functions specifically working memory which considers as a core component of executive functions. By exploring these relationships, we aim to contribute to the understanding of how physical and cognitive development are interconnected during this critical developmental period. Specifically, this research seeks to determine which aspects of physical fitness are most strongly related to different components of executive functions. This knowledge can inform the design of targeted interventions that simultaneously enhance physical fitness and executive functions, potentially leading to improved academic performance, better physical health, and enhanced social functioning in children.

## Method

### Participants

Participant boys aged 10–13 years from primary and middle schools from Taif city in Saudi Arabia were recruited to the study. Their families/parents were informed and invited to collaborate in the study. Before conducting the study, consent forms were provided for participants and their parents or guardians and schools. Ethical approval for the study was obtained from the King Saud University Ethical Review Committee (IRB: KSU-HE-24-234). We used G*Power software (V.3.1.9.7) to calculate required sample size to obtain 95% confident that our sample is representative of the population with a margin of error of 5%. a sample size of at least 99 participants was required, and we add 20% for any withdrawn and data missing. One-hundred thirty-seven children completed cognitive assessments and physical fitness tests, who were included in the analysis of current study. Exclusion criteria included age, any metabolic or mobility diseases, and not completing study requirements.

### Materials

#### Anthropometry and body composition

Participants’ heights and weights were measured to the nearest ±0.1 cm using a Seca model 220 portable stadiometer and to the nearest ±0.1 kg using a Seca 770 digital scale, respectively. Trained Saudi research assistants took all measurements. Body mass index (BMI) was calculated [weight (kg)/height^2^ (m^2^)] based on the CDC Global reference data (Cole et al., 1995), and participants classified as underweight, normal weight, overweight or obese according to IOTF criteria.

#### Physical fitness tests

**Shuttle run:** Cardiorespiratory fitness was assessed using the FITNESSGRAM PACER (Meredith and Welk, 2010) a modified version of 20 m shuttle-run test. The test is carried out inside or outside on a hard-court surface, and the 20 m distance was marked by two lines of standard cones. All participants follow a standardized recorded instruction from the PACER CD. The participants were instructed to run back and forth over the marked 20 m distance in time with the audible signal. The test speed starts with 8.5 km/h, with speed increment of 0.5 km/h every 1 min which represents a stage. The test is ended either at the point of individual volitional exhaustion or when participants failed to maintain the required running speed twice (Miguel-Etayo et al., 2014). Performance is quantified as total shuttles completed, and classification of aerobic fitness level reported by Tomkinson et al., has been used (Tomkinson et al., 2018). This test has been used in the Saudi adolescents (Aljuhani and Sandercock, 2019).

**Grip strength:** Strength was assessed using isometric handgrip dynamometry (Baseline® Smedley spring-type dynamometer, Fabrication Enterprises, Inc., White Plains, NY, USA). The dynamometer is adjusted accordingly, to accommodate differences in participant hand size and assure a comfortable grip position. Participants were given verbal encouragement to apply maximal effort and ‘squeeze as hard as possible’ for at least 2 s. The protocol and instructions were designed to reduce the occurrence of Valsalva’s maneuver, reduce the frequency and degree of shoulder elevation, and maintain a neutral neck position. Researchers visually monitored each attempt to prevent participants from pushing the hand or dynamometer against the outer thigh. Two trials were performed using the dominant hand and the highest score is recorded as peak grip strength (kg). In cases where the second measure is much higher than the first attempt, a third trial will be performed and the highest value used. Limb dominance will be determined by asking participants which hand they wrote with. This test has been used in the Saudi children (Alqahtani et al., 2023).

**Long jump:** The test examines the strength of lower limbs. The participant stands on a line at a start point, feet close to each other bending his/her knees and try to jump pushing the body to the front as much as possible. The connection between the heel and the ground is marked, and distance from the starting point is measured using a measuring tape. Participant is given to trials, and the highest is recorded in Castro-Piñero et al. (2009). Results are classified based on the reference values reported by Tomkinson et al. (2018). This test has been used in the Saudi children (Duncan et al., 2023).

#### Executive function tasks

The three tasks of executive functions were performed by the Inquisit web software which provides an access to all the tasks and also stores the data automatically. All the tasks can be opened and performed in tablets. All the tasks together take around 10 min.

**Figure 1:**
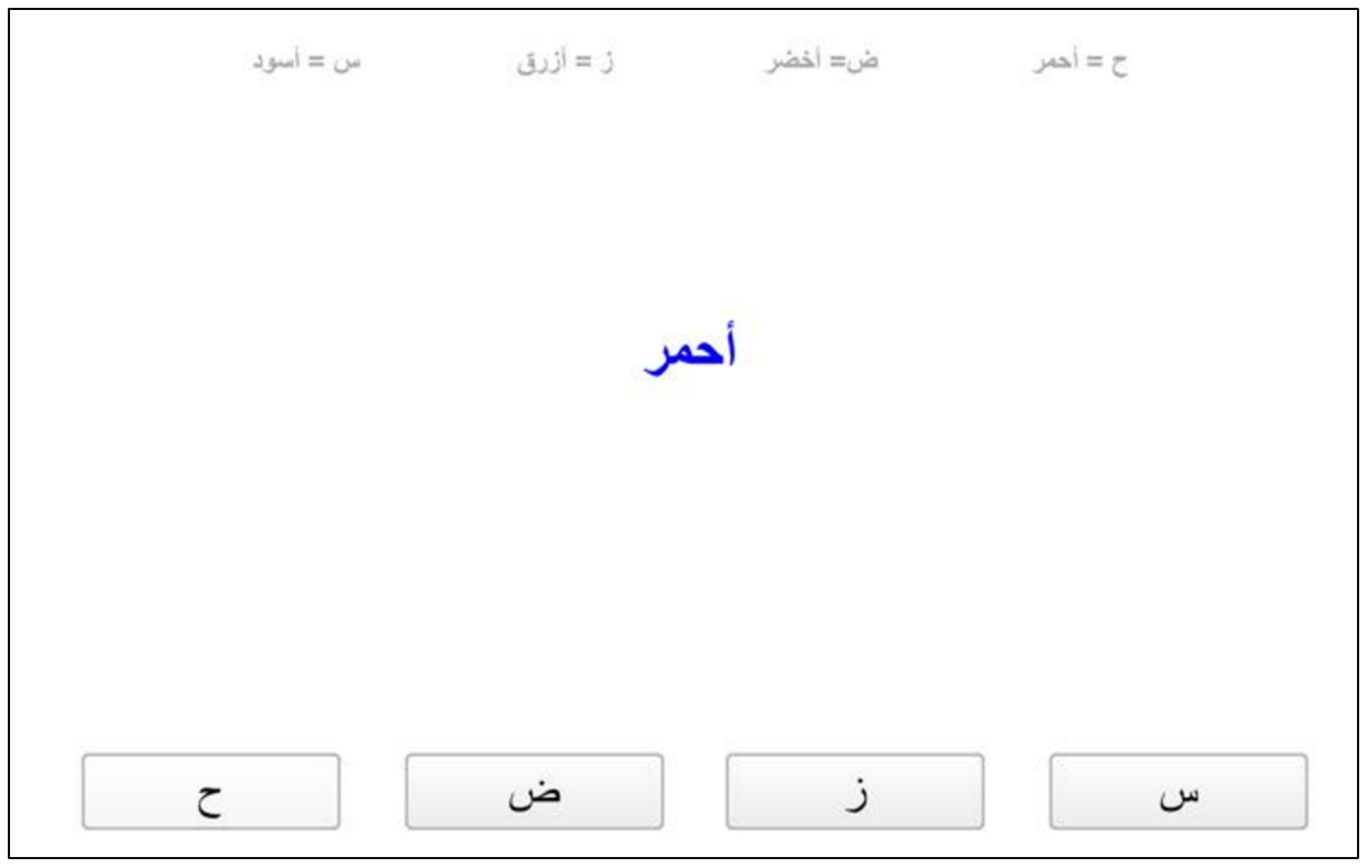
A single trail of Stroop task.

**Stroop (inhibition control):** is an Arabic version designed specifically for this study. In this task the participant has to choose the ink for the written color name in Arabic (red, green, black and blue). The participant has to press the assigned key for the ink of the color name regardless the meaning of the word. For example, the word Red is written in a blue ink, so the participant has to choose the assigned key for the blue ink (See Figure 1). The task starts with an instruction and practice trails to make sure the participants understand the requirement for the task. The mean accuracy and the mean reaction time are recorded in the software (Jensen and Rohwer, 1966).

**Figure 2:**
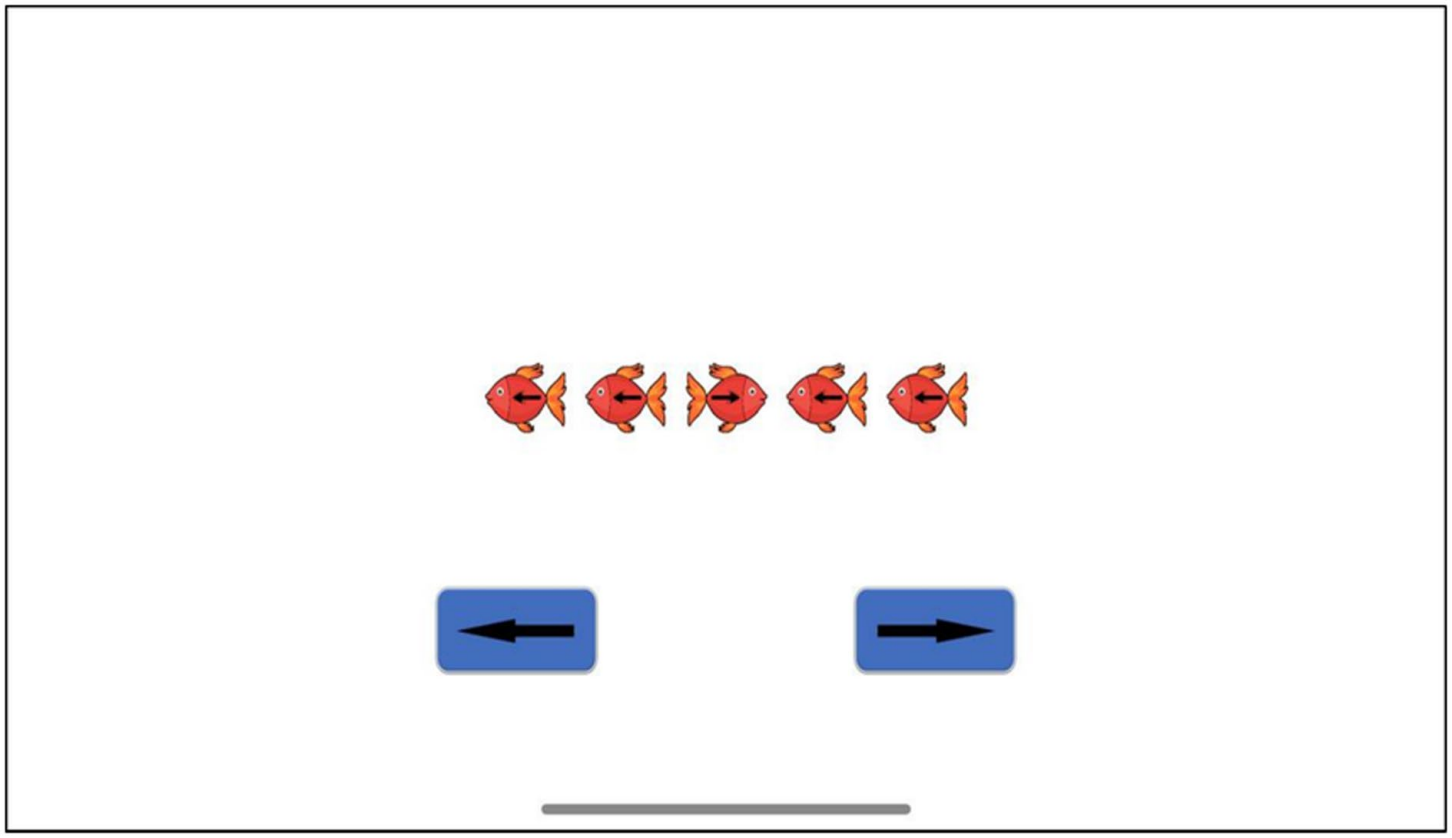
A single trial of Flanker task.

**Flanker (selective attention):** is a well-known and widely used task to assess selective attention and speed of information processing. Five arrows inside fish appear in the screen facing different directions, a participant is required to focus only on the central one and press the corresponded button in the screen (See Figure 2). For example, if the central arrow faces the right direction, then the participant has to press the right button on the screen. The accuracy rate (number of correct response) and the reaction time are recorded in the researcher’s platform.

**Figure 3:**
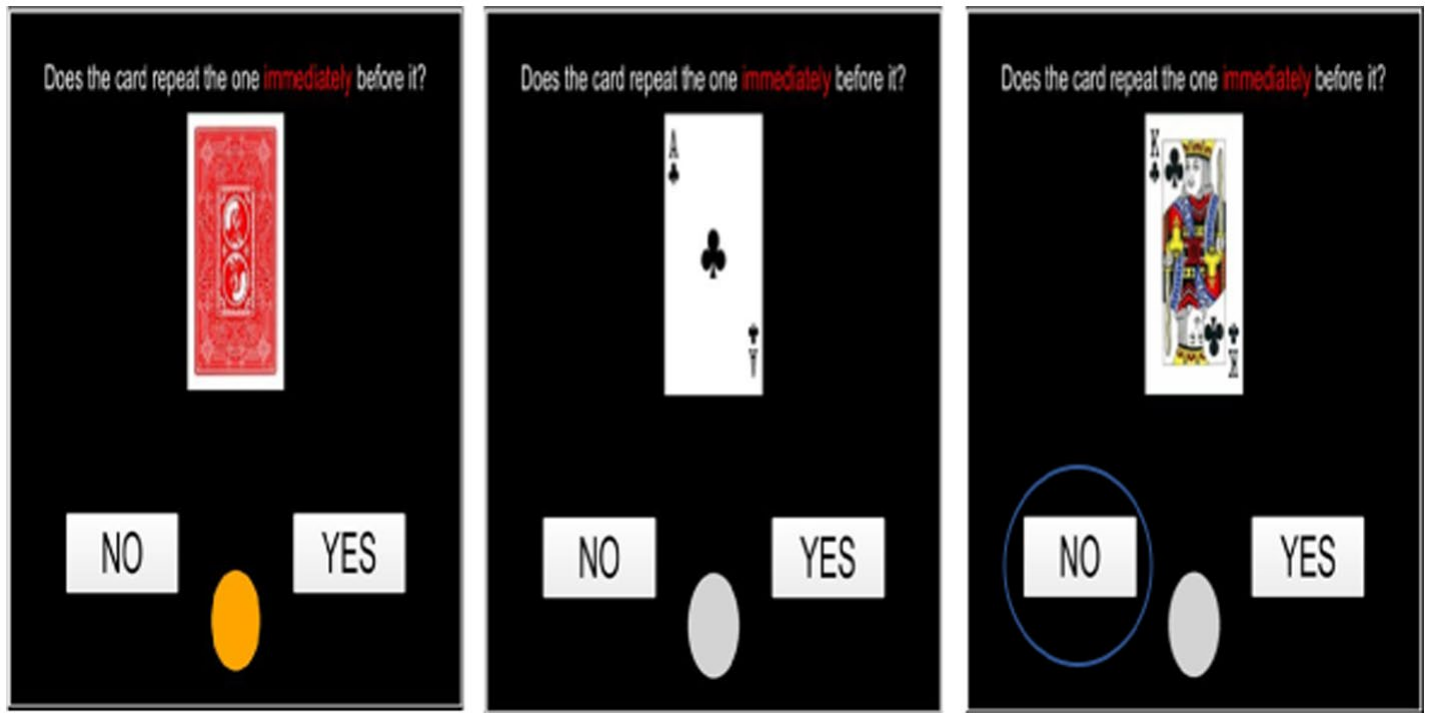
A trail of N.Back task where the previous stimuli is different and the participant has to choose No.

**N.Back (working memory):** the N-back task is a continuous performance task commonly used to assess working memory (Pelegrina et al., 2015). In this task, participants are presented with a sequence of stimuli (e.g., Playing Cards) and are required to indicate when the current stimulus matches the one presented N steps earlier in the sequence. If it is the same the participant has to select Yes and if it is not the same the participant has to select No (See Figure 3).

### Study design and procedure

The study was a cross-sectional design. Children who expressed their interests with the acceptance of their parents/gradients were included in the study. All tests were carried out in the morning between 8 and 10 am. In the first day, anthropometry and body composition measures were taken. Inquisit application is downloaded on a tablet, and all three executive function tasks were performed consequently, which is directly saved to the research account. These tests were performed in a private room. On the second day, handgrip strength and long jump tests were performed in the sport field outside the school building. Shuttle run test was performed in a separate day in the sport field. There was a minimum one researcher or research assistant for every 10 participants during the Shuttle run test.

### Data analysis

All data analyses were conducted using JASP, a user-friendly software for statistical analysis. Mean and standard deviation for age, executive function tasks, BMI and physical tests are presented in Table 1. Before performing the main analyses, we checked for outliers in our dataset to ensure the accuracy and reliability of our results. To explore the relationships between age, BMI, physical fitness tests (grip strength, long jump, and shuttle run), and executive function tasks (Stroop, N-Back, and Flanker), we conducted Pearson correlation analyses. To examine the specific contributions of physical fitness tests to working memory performance, assessed using the N-Back task, we conducted multiple linear regression analyses. In these analyses, age and BMI were included as control variables to isolate the effect of motor skills on working memory. By controlling for these covariates, we aimed to determine the unique contribution of each physical fitness tests (grip strength, long jump, and shuttle run) to working memory performance. Beyond age and BMI, factors such as nutritional status, family environment, socioeconomic status, and gender (not applicable in our single-gender study) can influence both physical fitness and cognitive outcomes. While our study design did not allow full control of these variables, future longitudinal or intervention-based projects should include more detailed assessments to isolate and examine these potential confounders. All analyses were conducted with an alpha of *p* < 0.05 level of significance.

**Table 1:** Mean and SD scores for the age, executive function tasks, BMI and physical fitness tests.

	Age	Stroop accuracy	Stroop RT	N.Back accuracy	N.Back RT	Flanker accuracy	Flanker RT	BMI	Grip strength	Long jump	Shuttle run
Mean	11.614	0.896	1,878.016	0.629	957.714	0.924	850.026	18.366	19.170	134.526	21.368
95% CI mean upper	11.806	0.913	2,007.801	0.664	989.385	0.941	880.578	19.128	19.955	139.002	23.466
95% CI mean lower	11.421	0.879	1,748.231	0.594	926.043	0.907	819.473	17.605	18.385	130.050	19.269
SD. Deviation	1.140	0.098	768.165	0.209	187.452	0.099	176.755	4.506	4.645	26.394	12.375

## Results

### Associations between physical fitness tests and executive function tasks

Table 2 presents the correlations among age, BMI, physical fitness tests, and executive function tasks in children aged 10–13 years. Significant correlations were observed between several variables, highlighting the relationships between motor and cognitive measures.

**Table 2:** Correlations among age, BMI, physical fitness tests and executive function tasks.

Measure	Age	BMI	Grip strength	Long jump	Shuttle run
1. Stroop accuracy	−0.043	0.183*	0.032	−0.117	−0.104
2. Stroop RT	−0.249**	−0.185*	−0.158	−0.160	−0.025
3. N.Back accuracy	0.433***	0.228**	0.312***	0.108	0.176*
4. N.Back RT	−0.439***	−0.026	−0.254**	−0.270**	−0.234**
5. Flanker accuracy	−0.039	0.026	0.044	0.159	0.173*
6. Flanker RT	−0.311***	−0.072	0.104	0.014	−0.030

BMI, Body Mass Index; RT, Reaction Time; Executive Function Tasks; Stroop (Inhibition Control and Cognitive Flexibility), Flanker (Selective Attention and Speed of Information Processing), N.Back (Working Memory) and Physical Fitness Tests; Grip Strength, Long Jump and Shuttle Run *p < 0.05, **p < 0.01, ***p < 0.001.

**Stroop accuracy** showed a positive correlation with BMI (*r* = 0.183, *p* < 0.05), indicating that higher BMI was associated with better Stroop task accuracy. However, no significant correlations were found with age, grip strength, long jump, or shuttle run.

**Stroop reaction time** was negatively correlated with age (*r* = −0.249, *p* < 0.01) and BMI (*r* = −0.185, *p* < 0.05), suggesting that older children and those with higher BMI had faster reaction times on the Stroop task. No significant correlations were found with grip strength, long jump, or shuttle run.

**N-Back accuracy**, a measure of working memory, was positively correlated with age (*r* = 0.433, *p* < 0.001), BMI (*r* = 0.228, *p* < 0.01), grip strength (*r* = 0.312, *p* < 0.001), and shuttle run performance (*r* = 0.176, *p* < 0.05), indicating that older children, those with higher BMI, greater grip strength, and better shuttle run performance had higher accuracy on the N-Back task.

**N-Back reaction time**, was negatively correlated with age (*r* = −0.439, *p* < 0.001), grip strength (*r* = −0.254, *p* < 0.01), long jump (*r* = −0.270, *p* < 0.01), and shuttle run performance (*r* = −0.234, *p* < 0.01), suggesting that older children and those with better motor performance had faster reaction times on the N-Back task.

**Flanker accuracy**, showed a positive correlation with shuttle run performance (*r* = 0.173, *p* < 0.05), indicating that better shuttle run performance was associated with higher accuracy on the Flanker task. No significant correlations were found with age, BMI, grip strength, or long jump.

**Flanker reaction time**, was negatively correlated with age (*r* = −0.311, *p* < 0.001), indicating that older children had faster reaction times on the Flanker task. No significant correlations were found with BMI, grip strength, long jump, or shuttle run.

These results highlight the intricate relationships between age, physical fitness, motor skills, and executive functions, suggesting that motor proficiency and physical health may contribute to cognitive performance in children.

### The contribution of physical fitness tests on executive function (working memory)

The multiple linear regression analyses were conducted to examine the contribution of various physical fitness tests to working memory performance (N-Back task) while controlling for age and BMI. The results are summarized in Table 3.

**Table 3:** The multiple linear regression analysis outcomes of physical fitness tests predicting working memory when controlling for age and BMI.

Dependent variable	Predictors	*β*	*t*	*p*	sr	*R*	*R*^2^	Adj. *R*^2^
N.Back	Age	0.531	5.612	<0.001	0.44	0.44	0.2	0.18
	Grip strength	−0.242	−0.2.559	0.012	−0.218			
	Age	0.48	5.849	<0.001	0.455	0.43	0.22	0.2
	Long jump	−0.256	−3.126	0.002	−0.263			
	Age	0.443	5.208	<0.001	0.414	0.43	0.17	0.16
	Shuttle run	−0.134	−1.581	0.116	−0.137			
	BMI	0.15	1.766	0.08	0.151	0.34	0.12	0.11
	Grip strength	0.268	3.163	0.002	0.264			
	BMI	0.318	3.567	<0.001	0.296	0.31	0.1	0.1
	Long jump	0.229	2.575	0.01	0.218			
	BMI	0.319	3.61	<0.001	0.299	0.34	0.12	0.1
	Shuttle run	0.299	3.382	<0.001	0.281			

BMI, Body Mass Index, according to Cohen’s guidelines, *r* ≥ 0.10, *r* ≥ 0.30, and *r* ≥ 0.50, represent small, medium, and large effect sizes, respectively (Cohen, 2013).

Regression analyses reveal that age and grip strength significantly predict working memory measured with N.Back task. The total contribution of age and grip strength to predicting working memory was 44%, with age accounting for 24% of the variance and grip strength adding 20% of the variation. Age and long jump are also significant predictors on working memory, with 43% total contribution and long jump accounts little more on the variance with 22%. The last physical fitness test shuttle run is not a significant predictor of working memory.

BMI is added in the second regression analyses to determine its contribution with physical fitness tests on working memory. When grip strength was included as a predictor along with BMI, it was found to be a significant positive predictor of working memory with 34% total contribution. BMI explains 22% of the variance and grip strength accounts for 12% of the variance. Long jump performance is also a significant positive predictor of working memory assessed with N-Back task when controlling for BMI. The total contribution of both variables 31% with long jump adding around 10% of the variation. Although shuttle run is not a significant predictor of working memory when age is controlled, shuttle run in the second model is predicting working memory when BMI is accounted for. The total contribution of BMI and shuttle run is 34% with shuttle run accounting for 12% of the variation.

These findings suggest that while age is the strongest predictor of working memory performance among children aged 10–13 years, certain physical fitness tests such as grip strength and long jump show a significant relationship with working memory performance. In contrast, shuttle run performance did not significantly predict working memory performance when age was controlled.

## Discussion

The present study aimed to investigate the associations between physical fitness and executive function tasks in children aged 10–13 years. Our findings reveal that the N-Back task, a measure of working memory, is significantly correlated with all three physical fitness tasks (grip strength, long jump, and shuttle run). These results are consistent with previous research highlighting the relationship between physical fitness and cognitive functions, particularly working memory in children and preadolescents (Chaddock-Heyman et al., 2014a,b; Hsieh et al., 2018; Serra et al., 2021; Wassenberg et al., 2005). The observed correlations suggest that higher physical fitness levels are associated with better working memory performance in children. This finding supports the notion that physical activities requiring strength, coordination, and endurance may enhance cognitive processes underlying working memory (Becht et al., 2020; Erickson et al., 2014; Erickson et al., 2011; Niederer et al., 2011; Van der Fels et al., 2015; Van Waelvelde et al., 2020). The present results also can be attributed to the hypothesis that significant striatal pathways exist between the cerebellum and the dorsolateral prefrontal cortex, which are crucial brain regions for both intricate movements and advanced cognitive functions (Diamond, 2000; Koziol et al., 2014; Leisman et al., 2016).

Interestingly, the Flanker task, which assesses selective attention, was only significantly correlated with the shuttle run task. This aligns with prior studies highlighting the importance of aerobic fitness, often measured through tasks like the shuttle run, in supporting attentional control (Chaddock et al., 2011; Donnelly et al., 2016; Páez-Maldonado et al., 2020). In a systematic review by Meijer et al. (2020), the findings indicated that in some studies there were insignificant improvements in physical fitness on selective attention assessed with the Flanker task between the intervention groups, which is in line with our findings. The absence of significant correlations between the Flanker task and grip strength or long jump may suggest that cardiovascular endurance, rather than muscular strength, plays a more critical role in enhancing selective attention (Amenya et al., 2021). For the Stroop task, which measures inhibition control, there was no significant correlation with the three physical fitness tests. This aligns with previous research suggesting that only certain aspects of cognitive functions are associated with specific components of physical fitness (Amenya et al., 2021; Haverkamp et al., 2021; Van der Fels et al., 2015; Van Waelvelde et al., 2020). The absence of an association between Stroop and the fitness tests highlights the complexity of EF and underscores the need to examine specific subcomponents of cognition (Amenya et al., 2021; Haverkamp et al., 2021). One explanation might be that inhibition control relies on distinct neural pathways that are less influenced by acute or moderate fitness levels, whereas working memory and selective attention share more overlapping neural substrates with motor function and cardiovascular endurance (Diamond, 2000; Koziol et al., 2014). Thus, while our results align with emerging studies that link physical activity to improved EF, they also emphasize that the relationship may be task-specific. This variation in associations was a key reason for selecting multiple cognitive tasks in this study, aiming to capture different dimensions of cognitive functions.

The second objective of this study was to determine the contribution of physical fitness tasks to working memory performance, assessed with the N-Back task, when controlling for age and BMI. The regression analyses demonstrated that physical fitness tasks had an additional significant contribution to working memory beyond the effects of age and BMI. This finding highlights the unique role of physical fitness in enhancing cognitive functions in children, independent of their developmental stage and body composition (Kao et al., 2017). Grip strength, long jump, and shuttle run each showed a significant positive contribution to working memory performance, suggesting that both muscular strength and cardiovascular fitness are crucial for cognitive development (Amenya et al., 2021; Caspersen et al., 1985; Chaddock-Heyman et al., 2014a,b; Chaddock et al., 2011; Donnelly et al., 2016; Drozdowska et al., 2021; Erickson et al., 2014; Erickson et al., 2011; Haverkamp et al., 2021; Kao et al., 2017; Anna Meijer et al., 2021; Niederer et al., 2011; Nieto-López et al., 2020; Páez-Maldonado et al., 2020). The positive relationship between these physical fitness tasks and working memory underscores the importance of incorporating diverse physical activities in children’s routines to foster both physical and cognitive health (Serra et al., 2021; Syväoja et al., 2021). Additionally, working memory is critically important for academic achievement in children, as it underlies essential cognitive processes such as problem-solving, reasoning, and comprehension (Gathercole et al., 2004; Alloway et al., 2009; Peng et al., 2016). Enhanced working memory capacity has been linked to better performance in subjects like mathematics and reading, highlighting its role in supporting learning and academic success (Barker, 2016; Gray et al., 2017; Kao et al., 2017; Syväoja et al., 2021). This was proven by Syväoja et al. (2021) when physical fitness and physical activities were associated significantly with math performance through working memory as a mediating factor in 12–17 years populations.

In terms of practical applications, there is growing evidence that integrating targeted physical activity programs into children’s daily routines can yield cumulative cognitive benefits (Amatriain-Fernández et al., 2021; Amenya et al., 2021; Bolger et al., 2021; Drozdowska et al., 2021; Gómez-Apo et al., 2021). If schools implement structured exercise sessions-encompassing both aerobic and strength exercises- children might experience lasting improvements in cognitive flexibility and working memory, essential skills for academic success. Indeed, consistent engagement in physical fitness routines over multiple years might reinforce neural adaptations, sustaining EF advantages into adolescence and beyond (Dallaway et al., 2023; Duncan et al., 2023; Nguyen et al., 2024). Schools and educators should consider promoting physical fitness programs that include strength training, aerobic exercises, and activities that enhance coordination. Such programs can potentially improve not only the physical health of children but also their cognitive functions, particularly working memory, which is critical for academic success (Barker, 2016; Gray et al., 2017; Kao et al., 2017; Syväoja et al., 2021). Similarly, similar findings might be utilized in the context of sports practice. Coaches and youth sports programs can enhance their training sessions by integrating activities that promote cardiovascular endurance (e.g., shuttle runs) alongside those that focus on muscular strength and motor abilities (e.g., grip strength and leaping drills). Integrated techniques may produce cognitive and physical benefits, hence endorsing a more comprehensive perspective of child development. Moreover, integrating strength training with cognitively challenging tasks—such as coordinating movements in reaction to fluctuating signals—can enhance and develop children’s executive skills (de Zarate et al., 2024; Syväoja et al., 2021). Consequently, training regimens in after-school programs or community sports clubs should priorities a combination of aerobic and anaerobic exercises to enhance children’s concentration, working memory, and overall cognitive abilities.

Our findings also resonate with neuroimaging literature suggesting that exercise-induced changes in the prefrontal cortex and cerebellum may underpin improvements in executive functions (Erickson et al., 2014; Leisman et al., 2016). While we did not directly measure neural activity, the observed correlations between specific physical fitness tests and working memory highlight a plausible link to those motor and cognitive brain regions (Diamond, 2000). Future research that incorporates functional MRI or EEG data could clarify these underlying mechanisms. Investigating neurobiological pathways, such as changes in brain structure and function due to physical activity, can provide deeper insights into how physical fitness enhances executive functions (Barenberg et al., 2011; Erickson et al., 2011; Niederer et al., 2011).

Considering the positives of the current study, which encompasses a rather extensive array of physical fitness and executive function measurements, certain limitations must be recognized. Our cross-sectional design limits the establishment of causality, and our sample—restricted to male children within a particular geographic area—may limit the generalizability of our results. Future longitudinal studies should aim for more diverse samples, including female children and participants from various socioeconomic and cultural backgrounds, to elucidate the directionality of these relationships and to investigate how specific training modalities may enhance executive function development over time.

In conclusion, this study offers additional evidence that several aspects of physical fitness are significantly correlated with executive functioning in children, especially working memory and selective attention. Recent research supports our findings, indicating that both aerobic fitness and muscular strength might positively affect children’s cognitive function, possibly through neurobiological mechanisms such as improved cerebral blood flow and elevated Brain-Derived Neurotrophic Factor BDNF levels. These findings highlight the necessity of advocating for varied, age-suitable physical activities—such as running, jumping exercises, and strength-training tasks—both within and outside educational environments.

By enhancing physical education curricula and promoting extracurricular sports, educators and policymakers can assist students in improving their physical health while simultaneously fostering vital cognitive abilities that facilitate academic success and daily functioning. However, additional comprehensive and long-term investigations are required to clarify causality, examine specific intervention methods, and enhance sample diversity. This research enables the formulation of evidence-based solutions that incorporate physical fitness into comprehensive educational and public health initiatives, thereby enhancing children’s well-being and performance both academically and socially.

## Data Availability

The raw data supporting the conclusions of this article will be made available by the authors without undue reservation.

## References

[ref1] Aljuhani O. Sandercock G. (2019). Contribution of physical education to the daily physical activity of schoolchildren in Saudi Arabia. Int. J. Environ. Res. Public Health 16:2397. doi: 10.3390/ijerph16132397, PMID: 31284531 PMC6651045

[ref2] Allom V. Mullan B. Hagger M. (2016). Does inhibitory control training improve health behaviour? A meta-analysis. Health Psychol. Rev. 10, 168–186. doi: 10.1080/17437199.2015.1051078, PMID: 26058688

[ref9002] Alloway T. P. (2009). Working memory, but not IQ, predicts subsequent learning in children with learning difficulties. Eur. J. Psychol. Assess. 25, 92–98.

[ref3] Alqahtani B. A. Alenazi A. M. Elnaggar R. K. Alshehri M. M. Alhowimel A. Najmi A. A.. (2023). Normative values for hand grip and pinch strength for 6 to 18 year-olds in Saudi Arabia. BMC Musculoskelet. Disord. 24:96. doi: 10.1186/s12891-023-06197-0, PMID: 36740670 PMC9899658

[ref4] Amatriain-Fernández S. Ezquerro García-Noblejas M. Budde H. (2021). Effects of chronic exercise on the inhibitory control of children and adolescents: a systematic review and meta-analysis. Scand. J. Med. Sci. Sports 31, 1196–1208. doi: 10.1111/sms.13934, PMID: 33559271

[ref5] Amenya P. C. A. Annan R. A. Apprey C. Kpewou D. E. (2021). Physical fitness and cognitive function among school–aged children in selected basic schools in the Ho municipality of Ghana. Heliyon 7:e06324. doi: 10.1016/j.heliyon.2021.e06324, PMID: 33732918 PMC7944041

[ref6] Barenberg J. Berse T. Dutke S. (2011). Executive functions in learning processes: do they benefit from physical activity? Educ. Res. Rev. 6, 208–222. doi: 10.1016/j.edurev.2011.04.002

[ref7] Barker L. A. (2016). Working memory in the classroom: an inside look at the central executive. Appl. Neuropsychol. Child 5, 180–193. doi: 10.1080/21622965.2016.1167493, PMID: 27191215

[ref8] Becht A. I. Klapwijk E. T. Wierenga L. M. van der Cruijsen R. Spaans J. van der Aar L.. (2020). Longitudinal associations between structural prefrontal cortex and nucleus accumbens development and daily identity formation processes across adolescence. Dev. Cogn. Neurosci. 46:100880. doi: 10.1016/j.dcn.2020.100880, PMID: 33202352 PMC7677671

[ref9] Bolger L. E. Bolger L. A. O’Neill C. Coughlan E. O’Brien W. Lacey S.. (2021). Global levels of fundamental motor skills in children: a systematic review. J. Sports Sci. 39, 717–753. doi: 10.1080/02640414.2020.1841405, PMID: 33377417

[ref10] Borghese M. M. Janssen I. (2019). Duration and intensity of different types of physical activity among children aged 10–13 years. Can. J. Public Health 110, 178–186. doi: 10.17269/s41997-018-0157-z, PMID: 30488347 PMC6964371

[ref11] Calvert S. Ross J. Hamlin M. (2001, 2001). Levels of physical activity of a sample of 10–13 year old New Zealand children. NZ Med J 114, 496–498. 11797874

[ref12] Caspersen C. J. Powell K. E. Christenson G. M. (1985). Physical activity, exercise, and physical fitness: definitions and distinctions for health-related research. Public Health Rep. 100, 126–131, PMID: 3920711 PMC1424733

[ref13] Castro-Piñero J. González-Montesinos J. L. Mora J. Keating X. D. Girela-Rejón M. J. Sjöström M.. (2009). Percentile values for muscular strength field tests in children aged 6 to 17 years: influence of weight status. J. Strength Cond. Res. 23, 2295–2310. doi: 10.1519/JSC.0b013e3181b8d5c1, PMID: 19826295

[ref14] Cerit E. Özlü K. Deryahanoglu G. Denizci T. Yamaner F. Kendirci H. N. P.. (2020). Determination of the basic motor skills and its relationship to BMI and physical activity level in preschooler. Afric. Educ. Res. J. 8, 115–123.

[ref15] Chaddock L. Pontifex M. B. Hillman C. H. Kramer A. F. (2011). A review of the relation of aerobic fitness and physical activity to brain structure and function in children. J. Int. Neuropsychol. Soc. 17, 975–985. doi: 10.1017/S1355617711000567, PMID: 22040896

[ref16] Chaddock-Heyman L. Erickson K. I. Holtrop J. L. Voss M. W. Pontifex M. B. Raine L. B.. (2014a). Aerobic fitness is associated with greater white matter integrity in children. Front. Hum. Neurosci. 8:584. doi: 10.3389/fnhum.2014.00584 25191243 PMC4137385

[ref17] Chaddock-Heyman L. Hillman C. H. Cohen N. J. Kramer A. F. (2014b). III. The importance of physical activity and aerobic fitness for cognitive control and memory in children. Monogr. Soc. Res. Child Dev. 79, 25–50. doi: 10.1111/mono.12129, PMID: 25387414

[ref18] Cohen J. (2013). Statistical power analysis for the behavioral sciences: Academic Press.

[ref19] Cole T. J. Freeman J. V. Preece M. A. (1995). Body mass index reference curves for the UK, 1990. Arch. Dis. Child. 73, 25–29. doi: 10.1136/adc.73.1.25, PMID: 7639544 PMC1511150

[ref20] Coull J. Frith C. Frackowiak R. S. J. Grasby P. (1996). A fronto-parietal network for rapid visual information processing: a PET study of sustained attention and working memory. Neuropsychologia 34, 1085–1095. doi: 10.1016/0028-3932(96)00029-2, PMID: 8904746

[ref21] Dallaway N. Lucas S. J. Ring C. (2023). Effects of Stroop task duration on subsequent cognitive and physical performance. Psychol. Sport Exerc. 68:102459. doi: 10.1016/j.psychsport.2023.102459, PMID: 37665903

[ref22] Dapp L. C. Gashaj V. Roebers C. M. (2021). Physical activity and motor skills in children: a differentiated approach. Psychol. Sport Exerc. 54:101916. doi: 10.1016/j.psychsport.2021.101916

[ref23] de Zarate A. E.-R. Powell D. Kühnhausen J. Allan J. L. Johnstone A. Crabtree D. R.. (2024). Free-living physical activity and executive function: a multi-study analysis of age groups and times of day. Int. J. Clin. Health Psychol. 24:100425. doi: 10.1016/j.ijchp.2023.100425, PMID: 38089542 PMC10714236

[ref24] Diamond A. (2000). Close interrelation of motor development and cognitive development and of the cerebellum and prefrontal cortex. Child Dev. 71, 44–56. doi: 10.1111/1467-8624.00117, PMID: 10836557

[ref25] Diamond A. (2013a). Executive functions. Annu. Rev. Psychol. 64, 135–168. doi: 10.1146/annurev-psych-113011-143750, PMID: 23020641 PMC4084861

[ref26] Diamond A. (2013b). “Want to optimize executive functions and academic outcomes? Simple, just nourish the human spirit” in Minnesota Symposia on child psychology: developing cognitive control processes: mechanisms, implications, and interventions (Hoboken, NJ, USA: John Wiley & Sons, Inc). PMC4210770 25360055

[ref27] Donnelly J. E. Hillman C. H. Castelli D. Etnier J. L. Lee S. Tomporowski P.. (2016). Physical activity, fitness, cognitive function, and academic achievement in children: a systematic review. Med. Sci. Sports Exerc. 48, 1197–1222. doi: 10.1249/mss.0000000000000901, PMID: 27182986 PMC4874515

[ref28] Drozdowska A. Falkenstein M. Jendrusch G. Platen P. Lücke T. Kersting M.. (2021). Interrelations of physical fitness and cognitive functions in German schoolchildren. Children (Basel) 8:669. doi: 10.3390/children8080669, PMID: 34438560 PMC8391688

[ref29] Duncan M. J. AlShabeb A. Fitton Davies K. Alshahrani N. Almasoud Y. (2023). A 6-week badminton-based movement intervention enhances fundamental movement skills and physical fitness in Saudi boys and girls. Sports 11:132. doi: 10.3390/sports11070132, PMID: 37505619 PMC10385236

[ref30] Erickson K. I. Leckie R. L. Weinstein A. M. (2014). Physical activity, fitness, and gray matter volume. Neurobiol. Aging 35, S20–S28. doi: 10.1016/j.neurobiolaging.2014.03.034, PMID: 24952993 PMC4094356

[ref31] Erickson K. I. Voss M. W. Prakash R. S. Basak C. Szabo A. Chaddock L.. (2011). Exercise training increases size of hippocampus and improves memory. Proc. Natl. Acad. Sci. USA 108, 3017–3022. doi: 10.1073/pnas.1015950108, PMID: 21282661 PMC3041121

[ref32] Gallahue D. L. (2010). Understanding motor development in children and youth. Proceedings of the 6th international scientific and expert symposium “contemporary views on the motor development of a child.

[ref9001] Gathercole S. E. Pickering S. J. Ambridge B. Wearing H. (2004). The structure of working memory from 4 to 15 years of age. Dev. Psychol., 40, 177. 14979759 10.1037/0012-1649.40.2.177

[ref33] Gómez-Apo E. Mondragón-Maya A. Ferrari-Díaz M. Silva-Pereyra J. (2021). Structural brain changes associated with overweight and obesity. J. Obes. 2021, 1–18. doi: 10.1155/2021/6613385, PMID: 34327017 PMC8302366

[ref34] Gray S. Green S. Alt M. Hogan T. Kuo T. Brinkley S.. (2017). The structure of working memory in young children and its relation to intelligence. J. Mem. Lang. 92, 183–201. doi: 10.1016/j.jml.2016.06.004, PMID: 27990060 PMC5157932

[ref35] Han X. Zhao M. Kong Z. Xie J. (2022). Association between fundamental motor skills and executive function in preschool children: a cross-sectional study. Front. Psychol. 13:978994. doi: 10.3389/fpsyg.2022.978994, PMID: 36092056 PMC9453748

[ref36] Haverkamp B. F. Oosterlaan J. Königs M. Hartman E. (2021). Physical fitness, cognitive functioning and academic achievement in healthy adolescents. Psychol. Sport Exerc. 57:102060. doi: 10.1016/j.psychsport.2021.102060

[ref37] Hsieh S.-S. Fung D. Tsai H. Chang Y.-K. Huang C.-J. Hung T.-M. (2018). Differences in working memory as a function of physical activity in children. Neuropsychology 32, 797–808. doi: 10.1037/neu0000473 30124313

[ref38] Jensen A. R. Rohwer W. D. Jr. (1966). The Stroop color-word test: a review. Acta Psychol. 25, 36–93. doi: 10.1016/0001-6918(66)90004-7, PMID: 5328883

[ref39] Kao S.-C. Westfall D. R. Parks A. C. Pontifex M. B. Hillman C. H. (2017). Muscular and aerobic fitness, working memory, and academic achievement in children. Med. Sci. Sports Exerc. 49, 500–508. doi: 10.1249/MSS.0000000000001132, PMID: 27776002

[ref40] Koziol L. F. Budding D. Andreasen N. D’Arrigo S. Bulgheroni S. Imamizu H.. (2014). Consensus paper: the cerebellum's role in movement and cognition. Cerebellum 13, 151–177. doi: 10.1007/s12311-013-0511-x, PMID: 23996631 PMC4089997

[ref41] Lamers M. J. Roelofs A. Rabeling-Keus I. M. (2010). Selective attention and response set in the Stroop task. Mem. Cogn. 38, 893–904. doi: 10.3758/MC.38.7.893 20921102

[ref42] Lee K. Bull R. Ho R. M. H. (2013). Developmental changes in executive functioning. Child Dev. 84, 1933–1953. doi: 10.1111/cdev.12096, PMID: 23550969

[ref43] Leisman G. Moustafa A. A. Shafir T. (2016). Thinking, walking, talking: integratory motor and cognitive brain function [review]. Front. Public Health 4:94. doi: 10.3389/fpubh.2016.00094, PMID: 27252937 PMC4879139

[ref44] Meijer A. Königs M. Vermeulen G. T. Visscher C. Bosker R. J. Hartman E.. (2020). The effects of physical activity on brain structure and neurophysiological functioning in children: a systematic review and meta-analysis. Dev. Cogn. Neurosci. 45:100828. doi: 10.1016/j.dcn.2020.100828, PMID: 32805531 PMC7451819

[ref45] Meijer A. Pouwels P. J. Smith J. Visscher C. Bosker R. J. Hartman E.. (2021). The relationship between white matter microstructure, cardiovascular fitness, gross motor skills, and neurocognitive functioning in children. J. Neurosci. Res. 99, 2201–2215. doi: 10.1002/jnr.24851, PMID: 34019710 PMC8453576

[ref46] Meredith M. D. Welk G. (Eds.). (2010). Fitnessgram and Activitygram Test Administration Manual-Updated 4th Edition. Human Kinetics.

[ref47] Miguel-Etayo D. Gracia-Marco L. Ortega F. Intemann T. Foraita R. Lissner L.. (2014). Physical fitness reference standards in European children: the IDEFICS study. Int. J. Obes. 38, S57–S66. doi: 10.1038/ijo.2014.136, PMID: 25376221

[ref48] Miyake A. Friedman N. P. Emerson M. J. Witzki A. H. Howerter A. Wager T. D. (2000). The unity and diversity of executive functions and their contributions to complex “frontal lobe” tasks: a latent variable analysis. Cogn. Psychol. 41, 49–100. doi: 10.1006/cogp.1999.0734, PMID: 10945922

[ref49] Mulder H. Pitchford N. J. Hagger M. S. Marlow N. (2009). Development of executive function and attention in preterm children: a systematic review. Dev. Neuropsychol. 34, 393–421. doi: 10.1080/87565640902964524, PMID: 20183707

[ref50] Nayfeld I. Fuccillo J. Greenfield D. B. (2013). Executive functions in early learning: extending the relationship between executive functions and school readiness to science. Learn. Individ. Differ. 26, 81–88. doi: 10.1016/j.lindif.2013.04.011

[ref51] Nguyen S. T. Guo J. Song S. Reyes-Dumeyer D. Sanchez D. Brickman A. M.. (2024). Physical activity moderates the relationship between cardiovascular disease risk burden and cognition in older adults. Neuroepidemiology 59, 1–11. doi: 10.1159/000536354, PMID: 38531336 PMC11424774

[ref52] Niederer I. Kriemler S. Gut J. Hartmann T. Schindler C. Barral J.. (2011). Relationship of aerobic fitness and motor skills with memory and attention in preschoolers (Ballabeina): a cross-sectional and longitudinal study. BMC Pediatr. 11, 1–9. doi: 10.1186/1471-2431-11-34, PMID: 21569343 PMC3107157

[ref53] Nieto-López M. Sánchez-López M. Visier-Alfonso M. E. Martínez-Vizcaíno V. Jiménez-López E. Álvarez-Bueno C. (2020). Relation between physical fitness and executive function variables in a preschool sample. Pediatr. Res. 88, 623–628. doi: 10.1038/s41390-020-0791-z, PMID: 32000261

[ref54] Páez-Maldonado J. A. Reigal R. E. Morillo-Baro J. P. Carrasco-Beltrán H. Hernández-Mendo A. Morales-Sánchez V. (2020). Physical fitness, selective attention and academic performance in a pre-adolescent sample. Int. J. Environ. Res. Public Health 17:6216. doi: 10.3390/ijerph17176216, PMID: 32867113 PMC7504082

[ref55] Pavlović S. Marinković D. Mitrović N. (2020). Motor skills of primary school children: the differences compared to age. doi: 10.5937/ZRPFU2022181P

[ref56] Pelegrina S. Lechuga M. T. García-Madruga J. A. Elosúa M. R. Macizo P. Carreiras M.. (2015). Normative data on the n-back task for children and young adolescents. Front. Psychol. 6:1544. doi: 10.3389/fpsyg.2015.01544, PMID: 26500594 PMC4597481

[ref9003] Peng P. Namkung J. Barnes M. Sun C. (2016). A meta-analysis of mathematics and working memory: Moderating effects of working memory domain, type of mathematics skill, and sample characteristics. J. Educ. Psychol., 108, 455.

[ref57] Pontifex M. B. Kamijo K. Scudder M. R. Raine L. B. Khan N. A. Hemrick B.. (2014). V. The differential association of adiposity and fitness with cognitive control in preadolescent children. Monogr. Soc. Res. Child Dev. 79, 72–92. doi: 10.1111/mono.12131, PMID: 25387416

[ref58] Serra L. Raimondi S. Di Domenico C. Maffei S. Lardone A. Liparoti M.. (2021). The beneficial effects of physical exercise on visuospatial working memory in preadolescent children. AIMS Neurosci. 8, 496–509. doi: 10.3934/Neuroscience.2021026, PMID: 34877401 PMC8611191

[ref59] Syväoja H. J. Kankaanpää A. Hakonen H. Inkinen V. Kulmala J. Joensuu L.. (2021). How physical activity, fitness, and motor skills contribute to math performance: working memory as a mediating factor. Scand. J. Med. Sci. Sports 31, 2310–2321. doi: 10.1111/sms.14049, PMID: 34519073

[ref60] Tomkinson G. R. Carver K. D. Atkinson F. Daniell N. D. Lewis L. K. Fitzgerald J. S.. (2018). European normative values for physical fitness in children and adolescents aged 9–17 years: results from 2 779 165 Eurofit performances representing 30 countries. Br. J. Sports Med. 52, 1445–1456. doi: 10.1136/bjsports-2017-098253, PMID: 29191931

[ref61] Tomporowski P. D. Davis C. L. Miller P. H. Naglieri J. A. (2008). Exercise and children’s intelligence, cognition, and academic achievement. Educ. Psychol. Rev. 20, 111–131. doi: 10.1007/s10648-007-9057-0, PMID: 19777141 PMC2748863

[ref62] Uttal D. H. Meadow N. G. Tipton E. Hand L. L. Alden A. R. Warren C.. (2013). The malleability of spatial skills: a meta-analysis of training studies. Psychol. Bull. 139, 352–402. doi: 10.1037/a0028446, PMID: 22663761

[ref63] Van der Fels I. M. Te Wierike S. C. Hartman E. Elferink-Gemser M. T. Smith J. Visscher C. (2015). The relationship between motor skills and cognitive skills in 4–16 year old typically developing children: a systematic review. J. Sci. Med. Sport 18, 697–703. doi: 10.1016/j.jsams.2014.09.007, PMID: 25311901

[ref64] Van Waelvelde H. Vanden Wyngaert K. Marien T. Baeyens D. Calders P. (2020). The relation between children's aerobic fitness and executive functions: a systematic review. Infant Child Dev. 29:e2163. doi: 10.1002/icd.2163, PMID: 40013540

[ref65] Vieira F. Fragoso I. Silva L. Castro L. C. (2003). Morphology and sports performance in children aged 10–13 years: identification of different levels of motor skills. Kinanthropometry VIII: Proceedings of the 8th international conference of the international society for the advancement of kinanthropometry (ISAK),

[ref66] Wassenberg R. Feron F. J. M. Kessels A. G. H. Hendriksen J. G. M. Kalff A. C. Kroes M.. (2005). Relation between cognitive and motor performance in 5- to 6-year-old children: results from a large-scale cross-sectional study. Child Dev. 76, 1092–1103. doi: 10.1111/j.1467-8624.2005.00899.x, PMID: 16150004

[ref67] Weinstein A. M. Voss M. W. Prakash R. S. Chaddock L. Szabo A. White S. M.. (2012). The association between aerobic fitness and executive function is mediated by prefrontal cortex volume. Brain Behav. Immun. 26, 811–819. doi: 10.1016/j.bbi.2011.11.008, PMID: 22172477 PMC3321393

